# Effect of Fluvoxamine on Carrageenan-Induced Paw Edema in Rats Evaluation of the Action Sites 

**Published:** 2011

**Authors:** Valiollah Hajhashemi, Hossein Sadeghi, Mohsen Minaiyan, Ahmad Movahedian, Ardeshir Talebi

**Affiliations:** a *Isfahan Pharmaceutical Sciences Research Center, School of Pharmacy and Pharmaceutical Sciences, Isfahan University of Medical Sciences, Isfahan, Iran.*; b *Department of Pharmacology, School of Pharmacy and Pharmaceutical Sciences, Isfahan University of Medical Sciences, Isfahan, Iran.*; c *Department of Pharmacology, School of Medicine, Yasuj University of Medical Sciences, Yasuj, Iran. *; d *Department of Biochemistry, School of Pharmacy and Pharmaceutical Sciences, Isfahan University of Medical Sciences, Isfahan, Iran.*; e *Department of Pathology, School of Medicine, Isfahan University of Medical Sciences, Isfahan, Iran.*

**Keywords:** Carrageenan, Fluvoxamine, Intra-cerebroventricular, Intra-thecal, Mifepristone, Rat

## Abstract

The present study was designed to explore the anti-inflammatory effect of fluvoxamine, as a selective serotonin reuptake inhibitor (SSRI) anti-depressant, on carrageenan-induced paw edema in more details. At first, fluvoxamine was administered intra-peritoneally (2.5, 12.5, 25 and 50 mg Kg^-1^) 30 min before the subplantar injection of carrageenan. Fluvoxamine was also injected intra-peritoneally at a dose of 50 mg Kg^-1^ 30 or 90 min after carrageenan injection. Then, fluvoxamine was given intra-cerebroventricularly (25, 50 and 100 μg/rat) and intra-thecally (25, 50 and 100 μg/rat) 30 min before the carrageenan challenge. Finally, the effect of mifepristone (5 mg Kg^-1^), an antagonist of the glucocorticoid receptor, on the anti-edema effect of fluvoxamine (50 mg Kg^-1^) was investigated. Results showed that intra-peritoneal (IP) administration of fluvoxamine before or after carrageenan injection considerably inhibited paw edema response at 4 h post-carrageenan (p < 0.001), but intra-cerebroventricular (i.c.v.) and intra-thecal (i.t.) injection of fluvoxamine did not alter the degree of paw swelling. The inhibitory effect of fluvoxamine was reduced by the pretreatment of mifepristone (p < 0.01).

Our results suggest that IP administration of fluvoxamine produces a noticeable anti-inflammatory effect in the carrageenan-induced paw edema in rats and at least, a part of this effect is mediated through glucocorticoid receptor. Moreover, it seems unlikely that central sites have an important role in this inhibitory effect of fluvoxamine.

## Introduction

Antidepressants are widely used in managing various types of pain such as neuropathic and inflammatory pains in addition to treatment of psychiatric disorders (1). In recent years, a compelling body of evidence has shown alterations of the activity of immune system in depressive patients (2, 3) and it has even been proposed that producing pro-inflammatory cytokines may have a crucial role in the etiology of depression due to the dysregulation of the immune system (4, 5). Therefore, if the immune system is involved in the etiology of depression, one would expect that antidepressant agents may exert some immunoregulatory effects. In this regard, several antidepressants such as fluoxetine, amitriptyline, trazodone, and desipramine have been reported to produce anti-inflammatory effects (6-9). Bupropion and fluoxetine have been also shown to exert some beneficial effects on the course of some inflammatory diseases such as Crohn›s disease and multiple sclerosis, respectively (10).

Selective serotonin reuptake inhibitors (SSRIs) drugs are broadly used in the treatment of depression, wide spectrum of anxiety disorders, and eating disorders. The reason is that the mentioned group of antidepressants has fewer side effects and is better tolerated in comparison with tricycle antidepressant drugs (TCAs). The analgesic effects of SSRIs have been shown in some experimental models of pain (11-14), but studies about their anti-inflammatory activities are limited and to some extent contradictory. Some SSRIs such as fluoxetine and doxepine exhibit the inhibitory effect on the carrageenan-induced paw edema, while Serteraline potentiates paw edema in rats (6, 15).

Fluvoxamine, a typical SSRI, showed analgesic effects in laboratory animals (11, 12); however, there is only one preliminary study available regarding the anti-inflammatory activity of fluvoxamine (15). Therefore, the aims of the current study were: (1) to extend our knowledge about the effect of IP administration of fluvoxamine on the carrageenan-induced paw edema, (2) to determine the potential involvement of central mechanisms in the anti-inflammatory activity of fluvoxamine by i.c.v. and i.t. administration of fluvoxamine, and (3) to evaluate the possible involvement of glucocorticoid receptor in this effect of fluvoxamine.

## Experimental

*Chemicals*

Fluvoxamine maleate was a gift from Abidi Pharmaceutical Co. (Tehran, Iran) and dissolved in a hydroalcoholic solution (70% isotonic saline; 30% ethanol). Carrageenan was purchased from Fluka Chemical (Switzerland) and dissolved in isotonic saline. Mifepristone and indomethacin (Sigma, St Louis, MO, USA) were suspended in aqueous carboxymethyl cellulose (2% w/v).

*Animals*

Male Wistar rats (200-250 g) were obtained from Animal House of Faculty of Pharmacy, Isfahan University of Medical Sciences, Iran. Rats were housed in standard polypropylene cages, four per cage, under a 12:12 h light and dark cycle with free access to food and water. Following surgical implantation of i.c.v. cannula, animals were housed one per cage to avoid possible dislocation of the cannula. The experiments were carried out in accordance with local guidelines for the care of laboratory animals of Isfahan University of Medical Sciences.

*Surgical procedures*

The animals were anesthetized with IP injection of ketamine (50 mg Kg^-1^) and xylazine (10 mg Kg^-1^) mixture. Then, rats were placed in a stereotaxic frame (Stoelting, USA) and an i.c.v. cannula was implanted with stereotaxic coordinates, AP: - 0.8 mm, L: 1.4 mm, V: 3.3 mm, according to Paxinos and Watson (16). To confirm the correct implantation of the cannula, all animals with i.c.v. cannulae were euthanized at the end of the experiments and the brain examined. Animals were handled daily 5 days before the experiment in order to acclimatize them to manipulation and minimize non-specific stress response.

*Intrathecal injection*

Drug injections at the lumbar level of the spinal cord were performed according to the method previously described by Mestre *et al. *(17). Briefly, the animals were gently controlled by hands of experimenter and a 27-gauge needle carefully inserted among L5-L6 spaces. A flick of the tail indicated that the spinal cord channel had been reached and the injection was given in a volume of 10 μL. As a rule, animals that did not show the reflex of tail at the first injection effort were discarded. 

*Carrageenan-induced paw edema*

The rats received a subplantar injection of 100 μL of 1% (w/v) suspension of carrageenan lambda in the right hind paw (18). The volume of the paw was measured by Plethysmometer (Ugo Basile, Italy) immediately before and then, 4 h after the carrageenan injection. Data were expressed as the increase in paw volume (mL) and compared with pre-injection values. 

*Experimental design*

The effect of IP injection of fluvoxamine (2.5, 12.5, 25 and 50 mg Kg^−1^, n = 8) on paw edema was studied in the first series of experiments. Fluvoxamine was given 30 min before subplantar injection of carrageenan. The control group received vehicle (n = 8; IP). Indomethacin (10 mg Kg^-1^, n = 8; IP) was used as a positive control. 

In the second series, the effect of IP injection of fluvoxamine on the course of established paw edema was studied. Fluvoxamine was given (50 mg Kg^-1^, n = 6; IP) 30 or 90 min after the carrageenan injection. The control group received vehicle (n = 6; IP) instead of fluvoxamine.

In the third series, we used the i.c.v. route to inject precise doses of fluvoxamine into the brain. The rats were given 5 days to recover from surgery. After that, fluvoxamine was administered smoothly within 1 min, via the injection cannula (25, 50 and 100 μg per rat) in a volume of 10 μL (n = 8). Instead of fluvoxamine, the control group received vehicle (n = 8; i.c.v.). 

In the forth series, we used the i.t. route to inject fluvoxamine into the spinal cord at the space between L5 and L6. Fluvoxamine was injected intra-thecally (25, 50 and 100 μg per rat, n = 6) 30 min prior to carrageenan in a volume of 10 μL. The control group received only i.t. injection of vehicle (n = 6). 

In the fifth series of experiments, in order to assess the possible involvement of the GC-receptor in the inhibitory effect of fluvoxamine, rats were pretreated with mifepristone (5 mg Kg^-1^, IP, a glucocorticoid antagonist; n = 6) 30 min prior to the IP injection of fluvoxamine (50 mg Kg^-1^). The control group was only subjected to mifepristone. 

*Statistical analysis*

Data were expressed as mean ± SD. The differences between vehicle control and treatment groups were tested by one-way analysis of variance (ANOVA) followed by the Tukey’s post-hoc test, using SPSS 13.0. The probability of p < 0.05 was considered to show the significant differences for all made comparisons.

## Results and Discussion

*Effect of IP injection of fluvoxamine on carrageenan-induced paw edema*

**Figure 1:**
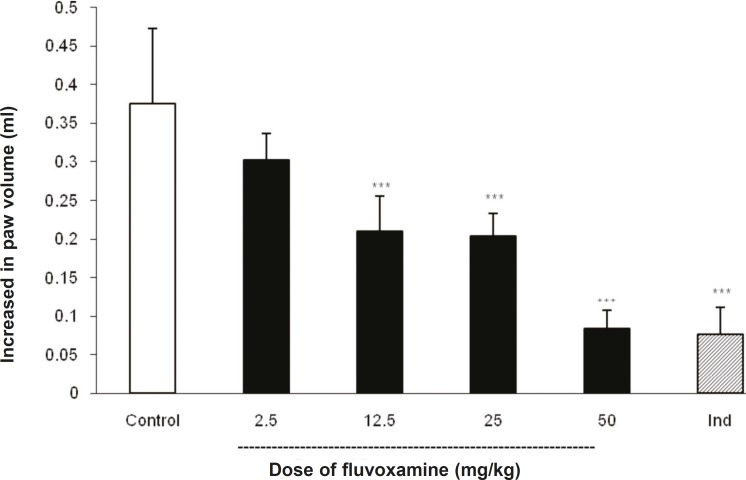
Effect of IP administration of fluvoxamine on the carrageenin-induced paw edema in the rat.

As illustrated in Figure 1, IP injection of fluvoxamine at doses of 2.5, 12.5, 25 and 50 mg Kg^-1 ^30 min before carrageenan, reduced paw swelling in a dose-dependent manner; although there was no marked difference in the responses produced at doses of 12.5 and 25 mg Kg^-1^. Post-hoc comparisons showed significant inhibition of edema formation by different doses of fluvoxamine at 4 h after the induction of inflammation (p < 0.001). As expected, the reference drug, indomethacin (10 mg Kg^-1^), also exhibited a significant inhibition of paw edema.

Fluvoxamine or the vehicle was administrated 30 min prior to the carrageenan (1%) injection and rats were evaluated for paw edema at 4 h post-carrageenan. The values represent the mean changes in the paw volume ± SD (n = 8, *** p < 0.001 compared with control group). Ind: indomethacin.

*Effect of IP injection of fluvoxamine on established inflammation*

**Figure 2:**
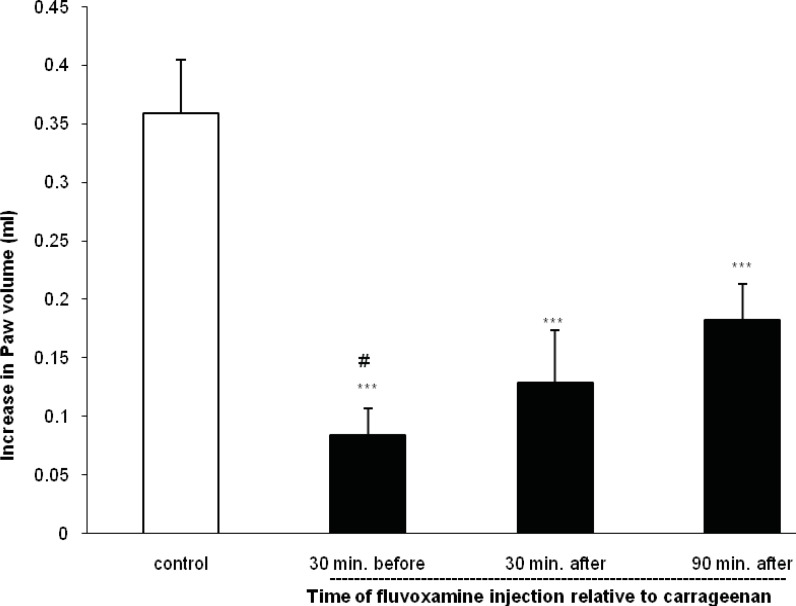
Effect of IP administration of fluvoxamine at a dose of 50 mg Kg^-1^ before (30 min) or after (30 and 90 min) carrageenan injection on the paw edema response at 4 h post-carrageenan

As shown in Figure 2, when fluvoxamine was given at a dose of 50 mg Kg^-1^ 30 or 90 min after carrageenan injection, it also inhibited further increase in the paw swelling (p < 0.001).

The values represent the mean increase in the paw volume ± SD (n = 6; ***: p < 0.001 compared with control group; #: p < 0.05 compared with 30 and 90 min after carrageenan).

*Effect of i.c.v. injection of fluvoxamine on carrageenan-induced paw edema*

**Figure 3:**
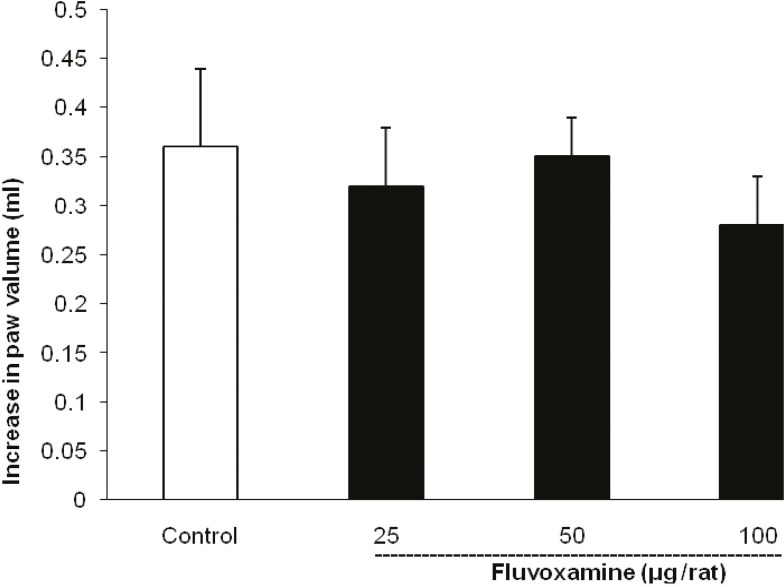
Lack of intra-cerebroventricular injection effect of fluvoxamine on the carrageenan-induced paw edema in the rat

As illustrated in Figure 3, the i.c.v. injection of fluvoxamine at doses of 25, 50 and 100 μg per rat, 30 min before carrageenan challenge, had no effect on the paw edema.

Fluvoxamine or the vehicle was administrated 30 min prior to the carrageenan (1%) injection and rats were evaluated for paw edema at 4 h post-carrageenan. The values represent the mean changes in the paw volume ± SD (n = 8).

*Effect of i.t. injection of fluvoxamine on carrageenan-induced paw edema*

**Figure 4:**
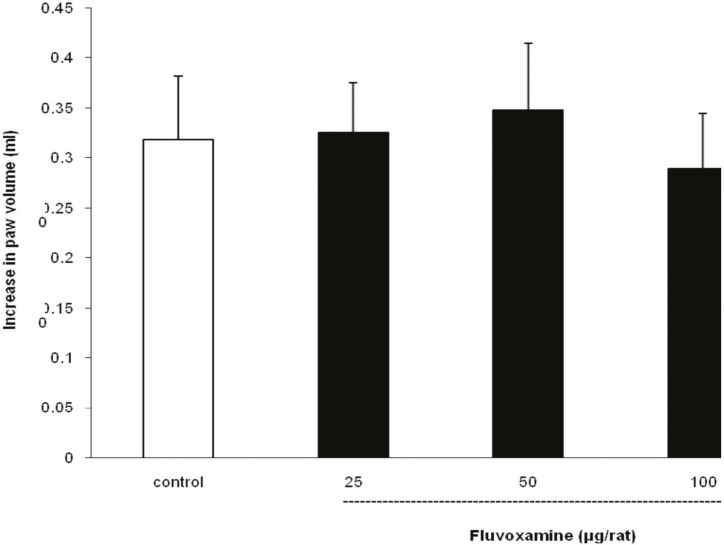
Lack of intra-thecal injection effect of fluvoxamine on the carrageenan-induced paw edema in the rat

As illustrated in Figure 4, i.t. application of fluvoxamine at doses of 25, 50 and 100 μg per rat, 30 min before carrageenan test, had no effect on peripheral inflammation induced by carrageenan.

Fluvoxamine or vehicle was administrated 30 min prior to the carrageenan (1%) injection and rats were evaluated for paw edema at 4 h post-carrageenan. The values represent the mean increase in the paw volume ± SD (n = 6).

*Effect of mifepristone on the anti-inflammatory effect of ip injected fluvoxamine*

**Figure 5:**
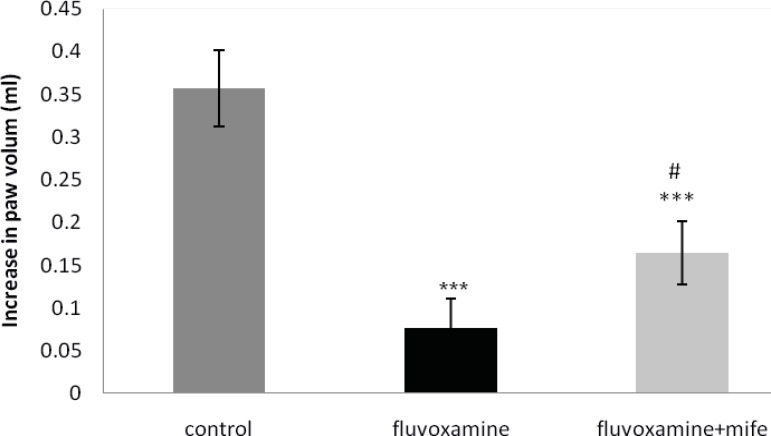
Effect of mifepristone on the anti-inflammatory activity of fluvoxamine (50 mg Kg^-1^).

Fluvoxamine (50 mg Kg^-1^, IP) significantly inhibited the carrageenan-induced paw edema. Pretreatment with mifepristone (5 mg Kg^-1^; IP; 30 min before fluvoxamine) considerably reversed the anti-inflammatory action of fluvoxamine (Figure 5).

The values represent the mean variation in the paw volume ± SD (n = 6. *** p < 0.001 compared with control group; # p < 0.01 compared with fluvoxamine group). mife: Mifepristone.

In the present study, we demonstrate that IP injection of fluvoxamine produces the marked anti-inflammatory effect in the carrageenan-induced paw edema. These results are consistent with the previous work done in our laboratory (15) and provide additional evidence for anti-inflammatory property of fluvoxamine. The effectiveness of fluvoxamine in reducing edema was dose-related and the value of inhibition at 50 mg Kg^-1^ dose was approximately equal to that observed with indomethacin (10 mg Kg^-1^). 

Manipulation of central levels of serotonin can affect the peripheral inflammation (19). Fluvoxamine could induce changes in the concentration of serotonin at the neural synoptic within the central nervous system by inhibiting serotonin reuptake (20, 21). In this regard, we sought to evaluate the possible role of central sites in the anti-edematogenic effect of fluvoxamine, but i.c.v. or i.t. injection of the drug at the indicated doses did not exhibit any inhibitory effect on the inflamed paw. These observations favor a direct peripheral mechanism for the anti-inflammatory activity of fluvoxamine and suggest that spinal and supraspinal mechanisms seem unlikely to have an important role in this action of fluvoxamine. It is worth mentioning that wide diversity actions of serotonin in the central sites may explain why we did not observe any considerable response after central application of fluvoxamine, due to multiple receptor subtypes (22). 

The mechanisms, by which antidepressants produce anti-inflammatory effect, are not clear. It has been shown that serotonin exerts a stimulatory effect on the pituitary-adrenocortical function in experimental animals (23, 24), and SSRIs may influence the activity of hypothalamo-pituitary-adrenal axis (HPA) apart from their effects on biogenic amine metabolism (25, 26). Since stimulation of HPA axis results in the secretion of cortisol, a well-known endogenous substance with strong anti-inflammatory property, from the adrenal cortex, we hypothesized that fluvoxamine may interact with glucocorticoid system to exert its anti-inflammatory effect. In this context, pretreatment with mifepristone significantly reversed the anti-inflammatory response of fluvoxamine, suggesting the involvement of glucocorticoid receptor in this effect of fluvoxamine. Indeed, it is the first work showing the role of glucocorticoid receptor in the anti-inflammatory effect of an antidepressant drug. Recent studies showed that antidepressants modulate GR function in brain and blood cells, by increasing GR translocation and increasing GR receptor expression; although variation in the ability of antidepressants to change GR expression and HPA axis function is obvious (27, 28). Based on the capacity of fluvoxamine in reducing inflammation, just when it was injected IP and revered this effect by a glucocorticoid receptor antagonist, it is plausible that fluvoxamine interacts with GR functions in some way. In other words, our results did not provide an interaction between fluvoxamine and HPA axis, but propose a clue for the role of GR in the anti-inflammatory effect of fluvoxamine. 

In conclusion, the acute carrageenan-induced inflammation is characterized by distinct phases and a number of mediators are involved in the inflammatory response of carrageenan. The initial phase observed around (0-1 h) is attributed to the release of histamine, serotonin, bradykinin and substance p, whilst the delayed phase (after 1 h) is mainly sustained by the migration of polymorphonuclear (PMN) cells to the inflammatory site which produce several pro-inflammatory mediators (29, 30). Since fluvoxamine reduced the paw edema when it was injected during the initial or the late phases (30 or 90 min after carrageenan injection), it is possible that fluvoxamine would have interacted with the PMN leucocyte infiltration or would have inhibited the release of PMN leukocyte-derived mediators. 

In brief, the results of present study provide further evidence for the anti-inflammatory effect of fluvoxamine. This effect seems mainly to be generated by a direct peripheral action of fluvoxamine, at least in part, by interaction with GC-receptor system. Furthermore, we suggest that the central sites do not have an important role in this action of fluvoxamine. Based on the potent anti-inflammatory effect of the drug, it may be a useful treatment in the management of inflammatory disease, especially in depressive 

patients who are suffering from inflammatory diseases such as Crohn’s, multiple sclerosis and rheumatoid arthritis.
